# The Structure of Compulsive Sexual Behavior: A Network Analysis Study

**DOI:** 10.1007/s10508-023-02549-y

**Published:** 2023-02-03

**Authors:** Igor Marchetti

**Affiliations:** Department of Life Sciences, Psychology Unit, University of Trieste, 34128 Trieste, Italy

**Keywords:** Compulsive sexual behavior, Network analysis, Impulsivity, Hypersexuality, ICD-11, DSM-5

## Abstract

Compulsive sexual behavior is a phenomenon characterized by a persistent failure to control intense, repetitive sexual impulses or urges, resulting in repetitive sexual behavior that causes marked distress or impairment in personal, familial, social, educational, or occupational areas of functioning. Despite its major impact on mental health and quality of life, little is known about its internal structure and whether this phenomenon differs across genders, age groups, and risk status. By considering a large online sample (n = 3186; 68.3% males), ranging from 14 to 64 years old, compulsive sexual behavior was explored by means of network analysis. State-of-the-art analytical techniques were adopted to investigate the pattern of association among the different elements of compulsive sexual behavior, identify possible communities of nodes, pinpoint the most central nodes, and detect differences between males and females, among different age groups, as well as between individuals at low and high risk of developing a full-blown disorder. The analyses revealed that the network was characterized by three communities, namely Consequence, Preoccupation, and Perceived Dyscontrol, and that the most central node was related to (perceived) impulse dyscontrol. No substantial differences were found between males and females and across age. Failing to meet one’s own commitments and responsibilities was more central in individuals at high risk of developing a full-blown disorder than in those at low risk.

**Supplementary Information:**

The online version contains supplementary material available at 10.1007/s10508-023-02549-y.

## Introduction

It has long been acknowledged that excessive sexual fantasies, urges, and behaviors may affect people’s lives and eventually impair their well-being and psychosocial functioning (Krafft-Ebing, 1907). A variety of terms, such as hypersexuality, sexual addiction, and out-of-control sexual behavior, have been used to describe this set of symptoms (Sassover & Weinstein, 2022). In 2019, compulsive sexual behavior disorder (CBSD) was officially recognized as a new diagnostic construct and included among the impulse-control disorders of the International Classification of Diseases 11th Revision (ICD-11; World Health Organization [WHO], 2019).

CSBD is defined as a persistent failure to control intense, repetitive sexual impulses or urges, resulting in repetitive sexual behavior that causes marked distress or impairment in personal, familial, social, educational, or occupational areas of functioning, for a period of at least six months. CBSD is excluded if the distress derives from moral judgements and disapproval about sexuality (WHO, 2019). Given its core elements, compulsive sexual behavior (CSB) consists of both observable features, namely frequent sexual activities and sex-related consequences, and subjective features, namely feelings that one’s sexual behaviors and thoughts are uncontrollable and the associated distress (Walton et al., 2017).

Individuals with CSB typically engage in non-paraphilic activities, namely masturbation, pornography, sex with anonymous partners, but they do so to the extent that their behavior substantially interferes with personal, interpersonal, and vocational occupations (Slavin, et al., 2020). CSB has been associated with a variety of negative consequences, such as sexually transmitted diseases, unwanted pregnancies, social isolation, reduced self-esteem, financial problems, and legal violations (Walton et al., 2017). Moreover, individuals with CSB often show comorbid disorders, namely mood disorders, anxiety disorders, substance abuse, personality disorders, and obsessive–compulsive disorders (Carpenter et al., 2013; Kafka & Hennen, 2002; Kingston & Firestone, 2008).

Although CSBD has been included in the ICD-11 as a new category (WHO, 2019), mounting evidence indicates that this phenomenon is organized dimensionally, along a continuum with increasing levels of sexual frequency and preoccupations (Graham et al., 2016; Walters et al., 2011). In other words, individual differences in CSB are better conceptualized as a matter of degree, ranging from low or negligible up to high and clinically relevant levels of frequent sexual behaviors and cognitions. Therefore, the current state of knowledge warrants research on nonclinical samples.

Several characteristics moderate the intensity of CSB. For instance, a recent systematic review reported that gender may play a significant role, with men typically reporting higher levels of CSB as compared to women (Kürbitz & Briken, 2021). Moreover, women are more worried than men about negative consequences of their sexual behavior, such as unwanted pregnancies, sexually transmitted diseases, physical injuries, and pain (Öberg et al., 2017). However, at least two features are worth considering. First, women may not report less frequent sexual intercourses than men (Långström & Hanson, 2006) and gender differences in reports of CSB may be due to stigma associated with women's sexual behavior (Baćak & Štulhofer, 2011; Lewczuk et al., 2017). Second, differences in CSB across gender are blurred when considering sexual orientation (Bőthe et al., 2018).

Less investigated is the relationship between CSB and age. Preliminary evidence shows that the age of onset of excessive sexuality in males is approximately 15 years old, with large variability (7–46 years) and median duration of 12 years (Kafka & Hennen, 2003). Both positive and negative correlations between age and CSB have been reported, although the magnitude of the effect is small (r <|.2|) (Dodge et al., 2004; Klein et al., 2015; Semple et al., 2006). This inconsistency in findings may be due to the fact that the majority of the studies recruited individuals within a limited age range, such as adolescents (Efrati & Gola, 2018), adults (Walton & Bhullar, 2018), or with comorbid conditions, such as elderly individuals with cognitive impairment (Wallace & Safer, 2009).

Despite the increasing interest in CSB, several characteristics of this phenomenon remain unexplored. First, it is largely unknown how the different features of CSB are specifically related to one another. Only one study has so far investigated the interaction of different elements of CSB (i.e., hypersexuality). By relying on network analysis (see below), Werner et al. (2018) revealed the central role of psychological distress due to one’s sexuality and sexuality-related negative feelings across both genders. Furthermore, sexual urge had a prominent role in men, whereas lack of control over sexual feelings had a prominent role in women. Hence, it is important to shed light on the specific network across the different features of CSB, as this could improve our understanding of the phenomenon (Borsboom & Cramer, 2013).

Second, several studies showed that men typically report higher mean levels of CSB than women do (Kürbitz & Briken, 2021, but see Långström & Hanson, 2006). However, it is unknown if the underlying network of associations among its elements also differ across genders. In other words, CSB could be structured in a similar fashion in men and women, but to a different degree of intensity. Werner and et al.’s (2018) study provided preliminary information about the associations among hypersexuality, negative consequences, and related sexual behaviors. They reported no significant difference across genders. Hence, further evidence on this topic is warranted.

Third, no strong evidence is currently available on CSB across different age groups. It is of importance to clarify if the structure of the associations among its elements changes across age or it persists in a stable fashion from adolescence up to late adulthood. On the one hand, time-limited periods of excessive sexual behavior have been reported, suggesting that CSB may be episodic and temporally unstable under certain circumstances (Kafka, 2010). On the other hand, there is evidence that CSB usually lasts for long periods of time (Kafka & Hennen, 2003). In sum, differences in CSB across different age groups are still unknown.

Fourth, given its dimensional nature (Graham et al., 2016; Walters et al., 2011), it is important to clarify if the structure of CBS differs between individuals at low or high risk to develop a full-blown disorder (i.e., CSBD). While the role of specific risk factors has been established (Grant Weinandy et al., 2022), it is unknown if the internal structure of CSB is different between individuals who show clinically relevant levels of CSB symptoms (i.e., high-risk) and those who do not report any substantial complaint (i.e., low-risk).

This study aims to reach four major goals. By capitalizing on previous literature (Werner et al., 2018), the first two objectives are to expand our understanding of CSB and to further explore gender differences. The second two objectives are novel: exploring how the network of CSB symptoms changes across different age groups and between individuals at low and high risk for developing CSBD.

To meet these goals, I adopted an innovative approach to understand psychopathology, namely network analysis (Borsboom & Cramer, 2013). This new perspective posits that clinical conditions (i.e., CSB) emerge from interactions among different types of thoughts, beliefs, and emotions, instead of being generated by an underlying factor (Borsboom & Cramer, 2013). In other words, while traditional approaches (i.e., factorial analysis) focus on which elements are more likely to cluster, network analysis explores how the different elements are related to one another (Bansal et al., 2020). By doing so, network analysis can show (1) all the links and their overall pattern (i.e., edges and network structure), (2) which elements are most strongly connected to all the other elements (i.e., node strength), (3) whether specific clusters of elements function in a similar way (i.e., communities), and (4) whether the network differs across groups (i.e., inter-network difference).

Two additional aspects of network analysis are worth commenting on. First, using estimating networks on cross-sectional data is contentious and open to various interpretations (Rodebaugh et al., 2018). In line with previous studies (Bernstein et al., 2019; Bottesi et al., 2020; Marchetti, 2019), in this study network analysis at the group level is intended to highlight the causal skeleton of CSB, where the edges between every pair of nodes represent links that can be directed, bidirectional, or influenced by unmodeled variables (Dalege et al., 2017). Subsequent idiographic (network) studies can then clarify the exact nature of these links at the individual level (Fisher et al., 2017). Therefore, the primary aim of this study is generating specific hypotheses about possible relationships among elements of CSB, which will be tested in subsequent experimental and longitudinal studies. Second, although network analysis is often applied to investigate specific psychological constructs (i.e., CSB), its domain is limited by the elements included in the analysis (i.e., questionnaire items) (Borsboom & Cramer, 2013). Despite this important limitation, recent meta-analytic evidence suggests that central nodes and robust edges are likely to emerge, even when different measures for the same construct are considered (Malgaroli et al., 2021).

In sum, I investigated the network structure of CSB in a large online sample of over 3000 individuals from adolescence to late adulthood, in order to reach the four goals of this study, namely (1) to establish the network structure of CSB, (2) to investigate if males and females differ with regard to the network structure, (3) to ascertain if the network structure changes across different age groups, and (4) to test if the network of CSB is different between individuals at low and high risk to develop a full-blown CSBD. To this end, I explored CSB as measured with the Sexual Compulsivity Scale (Kalichman & Rompa, 1995), which is one of the most frequently used self-report questionnaires for this phenomenon (Montgomery-Graham, 2017).

## Method

### Participants

Out of the initial sample of 3376 individuals, 191 individuals were excluded due to missing data, data entry errors, or inappropriate age-range. The final sample consisted of 3186 individuals (age 30.6 ± 10.9 years old; range = 14–64 years old; 68.3% males). Importantly, the sample included individuals from several age groups, namely adolescents (n = 128; age 14–17 years old; 60.9% males), young adults (n = 1195; age 18–25 years old; 59.00% males), adults (n = 1253; age 26–40 years old; 70.9% males), and older adults (n = 610; age 41–64 years old; 82.6% males). The data are publicly available from the Open-Source Psychometric Project (https://openpsychometrics.org/).^1^

### Measures

*Sexual Compulsivity Scale* (SCS; Kalichman & Rompa, 1995). The SCS is one of the most frequently used measures for assessing sexual compulsivity and it consists of 10 items, for which responses are given on 4-point Likert scale (i.e., 1 “Not at all like me”—4 “Very much like me”) (Hook et al., 2010). SCS has demonstrated excellent psychometric properties in terms of internal consistency (range Cronbach’s α = 0.77–0.90) and temporal stability over 2 weeks (*r* = 0.95; Kalichman & Rompa, 1995) and 3 months (*r* = 0.64, Kalichman et al., 1994). Several studies showed that SCS has strong convergent validity, in that SCS correlates with numbers of sexual partners, lower self-esteem, lower sexual control, and sexually transmitted diseases (Kalichman, 2020). SCS showed excellent concurrent validity with other measures of CSBD, such as the Hypersexual Behavior Inventory (*r* = 0.82; Reid et al., 2011) and the Hypersexual Disorder Screening Inventory (*r* = 0.87; Scanavino et al., 2016). Moreover, a systematic review of the literature showed that SCS has good construct validity, content validity, validity generalization, and internal consistency, as well as adequate norms (Montgomery-Graham, 2017). Previous research suggested a value ≥ 24 as a cutoff score for individuals at risk to develop a full-blown disorder (Montgomery-Graham, 2017, but see Ventuneac et al., 2015).

### Statistical Analysis

Initially, means and standard deviations of all the SCS items, in the whole sample and split by gender and age groups, were investigated. Further, I evaluated the informativeness of each item by means of standard deviation (Mullarkey et al., 2019) and explored the degree of redundancy among all the pairs of items (Jones, 2018).

Then, in accordance with the current guidelines (Epskamp & Fried, 2018), an EBIC graphical LASSO network model with all the SCS items was estimated, in order to obtain a sparse network consisting of non-spurious associations. Blue edges indicate positive associations, while red edges indicate negative ones, with saturation and thickness signifying stronger link between nodes.

In the context of network analysis, groups of nodes that are tightly linked and function in a rather similar way are defined communities (Fortunato, 2010). Although network communities and latent factors may be mathematically equivalent (Chandrasekaran et al., 2010), their interpretation differs substantially. On the one hand, latent factor analysis aims to identify the unobservable entity (i.e., CSBD) that generates the observable indicators (i.e., compulsive sexual behaviors, thoughts, etc.). On the other hand, the network approach contends that CSBD is generated by causal interactions among compulsive sexual behaviors, feelings and consequences (i.e., indicators). No latent, unobservable factor is needed (Costantini & Perugini, 2018; Dalege et al., 2016). In this study, community analysis was performed by means of the walktrap algorithm, which is largely used in psychological research (Golino & Epskamp, 2017).

Global network structure was explored with two indices, namely strength and predictability (Epskamp et al., 2018; Haslbeck & Waldorp, 2018; Jones, 2018). For each node, strength refers to the sum of the absolute weights of the edge (Valente, 2012), while predictability quantifies the amount of explained variance for a certain node by all the nodes connected to it (Hanslbeck & Waldorp, 2018).

Network accuracy and stability were investigated with a twofold approach, namely (1) centrality stability and bootstrapped difference test for the centrality index; (2) edge accuracy and bootstrapped difference test for edges (Epskamp et al., 2018). Strength indices and edges were considered stable if the correlation stability coefficient (i.e., *CS-coefficient*) was above 0.25, but preferentially above 0.5. The accuracy of the edges was investigated with 1000-bootstrap 95% nonparametric confidence intervals (CIs), with narrower CIs indicating that the estimated parameter is more reliable and trustworthy. Intra-network comparisons of strength indices and edges similarly relied on bootstrap CIs, with the difference being statistically significant if the CIs did not include zero.

In order to approximate how well the network structure could generalize to new data in independent studies, I integrated network estimation with tenfold cross-validation (James et al., 2013). Specifically, the association between every pair of nodes was quantified as “deviance explained” (R^2^_D_), namely the ratio of Kullback–Leibler divergence between fitted and null models (Cameron & Windmeijer, 1997). Importantly, R^2^_D_ approximates the traditional R^2^ and ranges from 0 to 1. Then, I iteratively estimated the pattern of associations on a subset of the whole sample (i.e., 9 out 10 folds; train subset) and subsequently tested it on data previously unseen by the model (i.e., 1 out of 10 folds; test subset). The procedure was repeated 10 times. Predictive deviance explained (predictive R^2^_D_) was estimated on the test subsets and indicates the fraction of uncertainty that the model is expected to account for in new data (Beevers et al., 2019; Marchetti et al., 2018). This analytical process was conducted by means of the R package “beset” (Shumake, 2018). Finally, partialization and regularization were applied on the matrix of predictive R^2^_D_ values, in order to compute an EBIC graphical LASSO network model. By doing so, I could build a cross-validated predictive network, which approximates to what extent the initial estimated network could generalize to new data and replicate in independent studies.

As for the inter-network comparison, I followed the statistical procedure outlined by van Borkulo et al. (2022) in order to investigate the network features between genders, age groups, and risk status. By relying on 1000 permuted data sets, each pair of networks (i.e., males vs. females; adolescents vs. adults, etc.; low-risk vs. high-risk) was tested with respect to the global connectivity difference (i.e., absolute sum of all the edges) and the network structure difference (i.e., maximum absolute element-wise difference). Depending on the latter, differences at the level of edges were also tested. Finally, strength differences between networks were evaluated.

## Results

### Descriptive Statistics

Total score, means, standard deviations, and Pearson’s correlations for the whole sample are reported in Table S1. Total score, means, and standard deviations split by genders, age groups, and risk status are shown in Tables S2, S3, and S4, respectively.

### Network Estimation, Community Detection, Network Inference, Stability, and Cross-Validation in the Whole Sample

The preliminary analysis revealed that no item was poorly informative (i.e. 2.5 SD below the mean level of informativeness; Mullarkey et al., 2019). Moreover, no item was detected as redundant with any other item (i.e., less than 25% statistically different correlations; Jones, 2018).

**Fig. 1:**
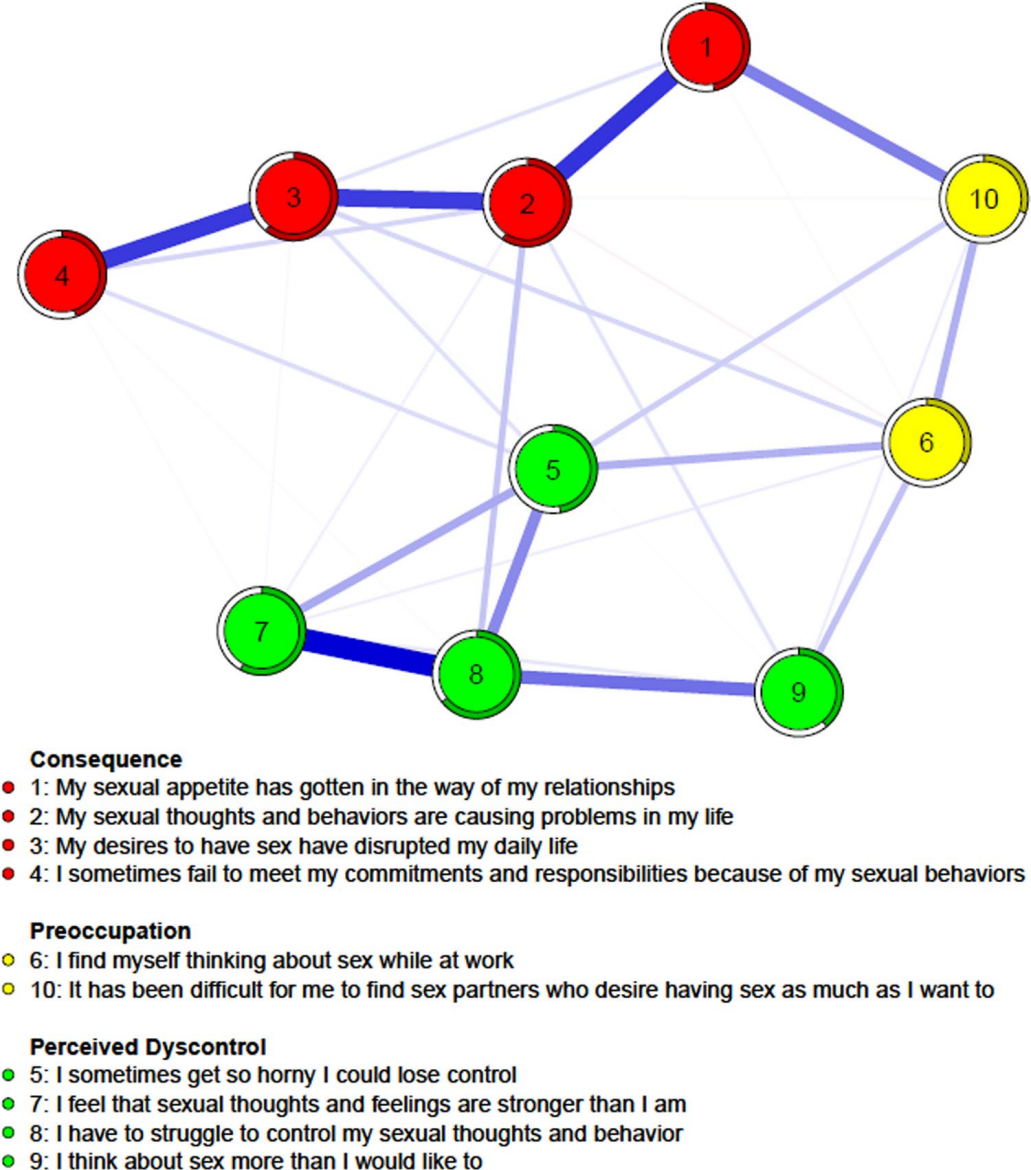
Network of CSB elements and community analysis

The network of cognitions and behaviors related to CSB across the whole sample is shown in Fig. 1, and several points are noteworthy. First, the analysis revealed a specific pattern of moderate interconnectedness among the different SCS variables (sparsity = 0.156), which was further qualified by the community analysis. The walktrap algorithm detected three communities, the first of which indicated the observable psychosocial consequences of CSB (“Consequence”), such as “My desires to have sex have disrupted my daily life” (item 3) and “I sometimes fail to meet my commitments and responsibilities because of my sexual behaviors” (item 4). The second community captured the subjective difficulties to control and manage sexual impulses (“Perceived Dyscontrol”), such as “I have to struggle to control my sexual thoughts and behavior” (item 8) and “I feel that sexual thoughts and feelings are stronger than I am” (item 7). Finally, a third community consisted of only two items, namely “I find myself thinking about sex while at work” (item 6) and “It has been difficult for me to find sex partners who desire having sex as much as I want to” (item 10). This last community captured variables indicating the contexts where CSB may occur in a problematic manner (“Preoccupation”).

The analysis of the edges revealed specific patterns of association. Within the Consequence community, the experience of strong sexual appetite as an obstacle to relationships (item 1) was associated with problems in one’s life due to sexual thoughts and behaviors (item 2), which were in turn linked with disrupted daily life (item 3). Eventually, experiencing interfered daily routine (item 3) was associated with failing to meet commitments and responsibilities (item 4). Within the Preoccupation community, thinking of sex while at work (item 6) was associated with the difficulties in finding a sexual partner who shows the same level of sexual desire (item 10). Interestingly, the Perceived Dyscontrol community mainly consisted of spending an excessive amount of time thinking about sex (item 9), which was associated with struggling to control sexual thoughts and behaviors (item 8) as well as feelings being overpowered by sexual impulses (item 7). The latter two items were also linked with the feeling of losing control due to sexual tension (item 5).

**Fig. 2:**
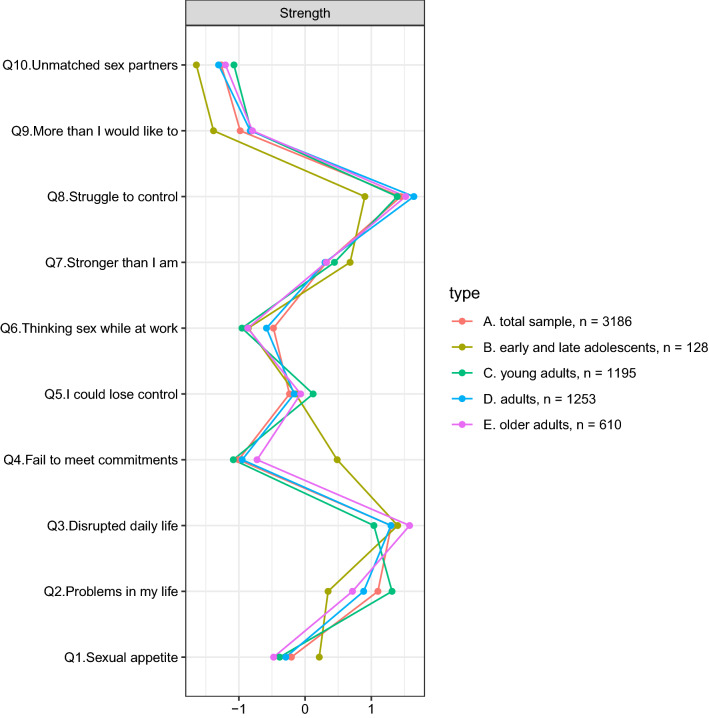
Strength indices in the whole sample and across adolescents, young adults, adults, and older adults

Importantly, the estimation of the edges was carried out in a very precise and stable way (Figure S1; CS-coefficient = 0.75), with the majority of the edges being statistically different from one another (Figure S2). The analysis of the network structure across the whole sample showed that four nodes had the highest centrality (Fig. 2), namely struggling to control sexual impulses and experiencing overwhelming sexual feelings (items 8 and 7; Perceived Dyscontrol community), going through a disrupted daily life and reporting problems in personal life (items 3 and 2; Consequence community). These four items were statistically stronger than the rest of the items (Figure S3) and the strength indices were estimated in a very stable way (CS-coefficients = 0.75). Moreover, the estimated network and the cross-validated predictive network were highly similar (Figure S4) and were associated with almost identical strength (Figure S5; *r*_s_ = 0.94). Hence, stability analysis and cross-validation suggest that the network is trustworthy and likely to generalize to new data.

The predictability analysis revealed that, on average, 48% of each item variance could be accounted for by the surrounding items, with the predictability indices ranging from 30 and 64%. Specifically, the Consequence and Perceived Dyscontrol communities could be explained to a degree of 53% and 52% of variance, respectively, while only 32% of the variance of the Preoccupation community could be accounted for.

### Network and Mean Level Differences by Gender

The networks of CSB in males and females (Figure S6) were highly correlated in terms of edges (*r*_s_ = 0.84) and strength indices (*r*_s_ = 0.92; Figure S7). The two networks were computed in a reliable and stable way (all CS-coefficients = 0.75; Figure S8). Similar to the network for the whole sample, struggling to control sexual thoughts and behaviors (item 8) was the most central item (Figure S5). Community analysis in the female group produced results similar to those of the whole sample, with disruptive sexual urges (item 1) and intrusive thoughts at work (item 6) clustering with the Preoccupation and Perceived Dyscontrol communities. In males, only the Consequence and the Perceived Dyscontrol communities were detected. Moreover, there was no difference in global connectivity (difference = 0.31, *p* = 0.09), while the network structure differed across gender (difference = 0.14, *p* < 0.02). Specifically, the edges between items 3 and 1, and items 1 and 6, were statistically stronger in males than in females. The edges between items 3 and 6, items 1 and 8, items 6 and 9, and items 1 and 10 were statistically stronger in females than in males. While all the differences were negligible (difference < 0.10), only the link between reporting sexual urges interfering with personal relationships (item 1) and having difficulty in finding a matching partners (item 10) was of small magnitude (difference = 0.11). At the level of node strength, item 1 was statistically stronger in females than in males (difference strength = 0.19, *p* < 0.017). At the level of mean levels, females reported higher levels of feeling of losing control due to sexual desire (difference = 0.23, *p* < 0.001), although the magnitude of the effect was small (Cohen’s d = 0.21; Table S2).

### Network and Mean Level Differences by Age

**Fig. 3:**
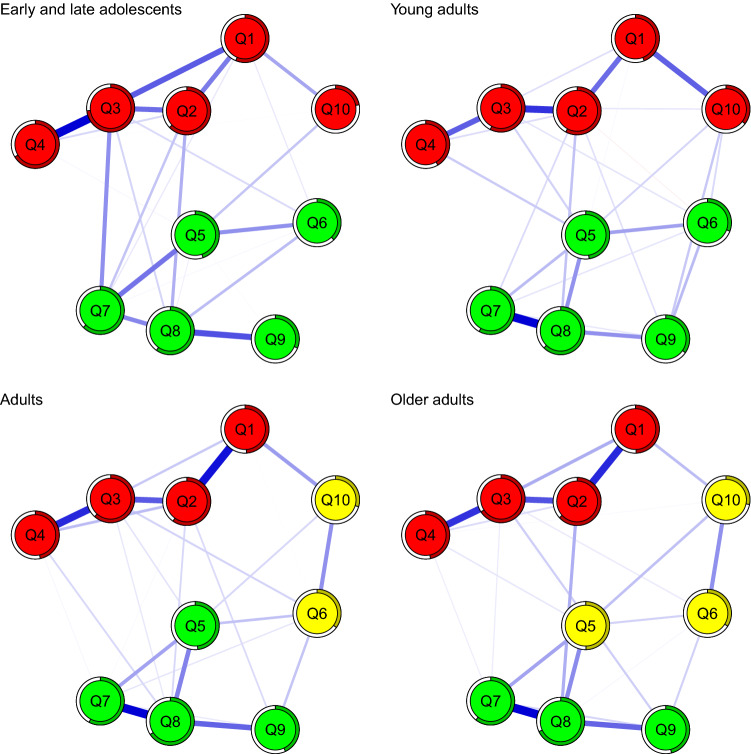
Network of CSB elements and community analysis across adolescents, young adults, adults, and older adults

The network of CSB was computed across the different age groups (Fig. 3). All the networks could be estimated in a reliable and stable way (Figure S9; CS-coefficient range = 0.44–0.75).

The networks across the four age groups were moderately to highly correlated, in terms of edges (range *r*_s_ = 0.67–85) and strength indices (range *r*_s_ = 0.64–96; Fig. 2) (Table 1). In a similar fashion across the age groups, struggling to control sexual thoughts and behaviors (item 8) and experiencing a disrupted daily life (item 3) were the most central item. Moreover, no difference at the level of global connectivity (all *p* = 1), network structure (range *p* = 0.72–1), or strength difference (range *p* = 0.18–1) was detected. Similarly, when age was entered as a moderator within the network (moderated network analysis; Haslbeck et al., 2021), no significant interactions were detected (Figure S10).

**Table 1:** Internetwork similarity indexes and network comparison tests adolescents, young adults, adults, and older adults

Comparison	Edge weights similarity	Strength similarity	Global connectivity difference	Network structure difference
ADO—YA	*r*_s_ = 0.73	*r*_s_ = 0.79	*p* = 1	*p* = 1
ADO—ADU	*r*_s_ = 0.67	*r*_s_ = 0.85	*p* = 1	*p* = .72
ADO—OA	*r*_s_ = 0.69	*r*_s_ = 0.85	*p* = 1	*p* = 1
YA—ADU	*r*_s_ = 0.85	*r*_s_ = 0.92	*p* = 1	*p* = .96
YA—OA	*r*_s_ = 0.80	*r*_s_ = 0.90	*p* = 1	*p* = 1
ADU—OA	*r*_s_ = 0.80	*r*_s_ = 0.95	*p* = 1	*p* = 1

*ADO* adolescents; *YA* young adults; *ADU* adults; *OA* adults

Spearman correlations (r_s_) were computed

*p*-values were adjusted for Bonferroni–Holm correction

When adolescents and young adults were considered, nodes were clustered in almost an identical fashion. The community analysis revealed only two clusters, namely Consequence and Perceived Dyscontrol. In adults and older adults, a similar pattern emerged, although the items representing the Preoccupation community (items 6 and 10), along with the feeling of losing control because of the intense sexual desire (item 5) in older adults, were clustered separately.

When investigating differences at the mean level across the four samples, four items were statistically different, namely items 1, 2, 5, and 10 (Table S3). However, effect sizes indicated small to negligible magnitude (η^2^ < 0.012). Finally, age was associated with any of the SCS items to a negligible degree (r =|.08|).

### Network and Mean Level Differences by Risk Status

By applying the standard cutoff for assessing the risk status (i.e., SCS score ≥ 24), 51.2% of the whole sample was classified at low risk and 48.8% at high risk. It is mentioning that sample splitting based on median or specific cutoff scores has been adopted in previous network analysis studies (McNally et al., 2014; Semino et al., 2017; Siew et al., 2019). The network of CSB was estimated in individuals at low or high risk (Figure S11), in a reliable and stable manner (Figure S12; CS-coefficient = 0.75 for both networks). Although the two networks were highly correlated in terms of edges (*r*_s_ = 0.85) and strength centrality (*r*_s_ = 0.83), they showed partially different communities. In particular, while the network in individuals at high risk was identical to that of the whole sample, individuals at low risk showed only two communities, namely Preoccupation and Perceived Dyscontrol.

Moreover, the two networks did not differ with respect to global connectivity (*p* = 0.69) and network structure (*p* = 0.14). However, the item reflecting the experience of failing to meet the commitments due to the interference of sexual behaviors (item 4) was statistically more central in individuals at high risk than in those at low risk to develop CBSD (*p* < 0.001; Figure S13). Finally, all the item means were largely higher in individuals at high risk than in those at low risk (Table S4).

## Discussion

CSB is characterized by a persistent failure to control intense, repetitive sexual impulses or urges, resulting in repetitive sexual behavior that causes marked distress or psychosocial impairment (WHO, 2019). Despite the increasing interest in this phenomenon, it has so far remained how the different components of CSB are specifically related to one another and if the pattern is stable across gender, age groups, and risk status. To bridge this gap, I performed a network analysis on a large sample, in order to shed light on the internal structure of this phenomenon.

Community analysis of the CSB network revealed the presence of three clusters, namely Consequences, Preoccupation, and Perceived Dyscontrol. The first community captured the negative outcomes of CSB on everyday life, relationships, and commitments, which have been extensively documented in the scientific literature. For instance, individuals with CSB often report serious problems due to their sexual activities, among which dropping out of school, losing jobs, unwanted financial losses, social isolation, marital adversities, and mental health distress (Koós et al., 2021). The second community consisted of only two items and referred to concerns that are frequent in the context of CSB, such as having sexual thoughts in workplace (Reid & Wolley, 2006) and the difficulty of finding a partner with similar levels of sexual drive (Hentsch-Cowles & Brock, 2013). Finally, the last community included items on perceived dyscontrol of sexual impulses and urges, which are later discussed in detail.

An almost identical clusterization of the items was found in a recent study that relied on exploratory factor analysis (EFA; Kingston et al., 2018). In my study, item 3 was included in the Consequence community, while in Kingston et al.’s (2018) study it saturated on the Perceived Dyscontrol factor. This result, however, is not unexpected, given that in the EFA study item 3 cross-loaded on both the Consequences and the Dyscontrol factors. Moreover, a partially similar structure was found for the Hypersexual Behavior Inventory (Reid et al., 2011), where the subscales Control and Consequences closely mirror the network communities Perceived Dyscontrol and Consequences. Hence, previous literature confirms the trustworthiness of the community analysis and strongly indicates that the higher-level structure of the Sexual Compulsivity Scale is characterized by three major areas.

When examining the network pattern of associations, specific nodes and edges are worth commenting on. Within the Consequence community, items 2 and 3 emerged as central nodes, indicating that in the context of CSB negative consequences are likely to be influential. In particular, sex-related psychosocial problems often take the form of failing to meet important commitments in the work place (i.e., edge between items 3 and 4) or experiencing disruptiveness in personal relationships (i.e., edge between items 2 and 1). In a recent study on over 5000 individuals (Koós et al., 2021), these two domains showed different profiles, in that having work problems was markedly associated with experiencing overall negative consequences, while having relationship problems was positively correlated with subjectively reported control impairments.

Within the Preoccupation community, experiencing relationship problems was linked with difficulties in finding a sex partner who matches the same intensity of sexual desire (i.e., edge between items 1 and 10). Previous evidence showed that members of couples likely differ with regard to their level of CSB (Starks et al., 2013), with intensity of relational problems being associated with the number of sexual and casual sexual partners (Koós et al., 2021). Taken together, these findings suggest a pattern of social deterioration, characterized by frequent and potentially conflicting sexual relationships.

Furthermore, experiencing sexual thoughts while being at work (item 6) was associated with both excessively frequent sexual fantasies and being preoccupied to lose control (i.e., edges between items 6 and 9 and items 6 and 5). In keeping with this, intensity of sexual desire and sexual urge were found to be linked with having problems at the workplace (Werner et al., 2018). Moreover, sex-related disruptions in the professional domain may lead to severe negative consequences, such as occupational problems (i.e., being fired; Schultz et al., 2014) and feelings of guilt and shame (Reid, 2010).

Within the Perceived Dyscontrol community, the most central node was struggling to control one’s own sexual thoughts and behavior (item 8), which was also the strongest node of the whole network. This finding confirms previous empirical and theoretical work that conceives of CSB as an impulsive disorder, according to which failing to resist an impulse for sexual activity plays a crucial role (Barth & Kinder, 1987; WHO, 2019).

In this study, the CSB network was substantially similar in men and women, with only negligible-to-small differences. A previous network analysis studies on hypersexuality reported no significant differences between genders (Werner et al., 2018), and measurement invariance between men and women has often been reported across measures of hypersexuality and CSB (Bőthe et al., 2018; Koós et al., 2021). It is to note that the difficulty to find a partner with similar sexual desire was more central in women than in men. A possible explanation may be that it is harder for women with CSB to reveal their intense sexual desires to their partner, in that feelings of shame are particularly powerful and disempowering in this group (Dhuffar & Griffiths, 2014; Ferree et al., 2012).

Importantly, the network of CSB elements was markedly stable when considering individuals between 14 and 64 years old. This is in line with evidence showing that specific features of human sexuality do not substantially change across the lifespan. For instance, CSB has been positively associated with “sexual excitation proneness” and negatively linked with “sexual inhibition due to possible threatening consequences” (Miner et al., 2016; Rettenberger et al., 2016). Both these features have been considered as stable traits that are largely genetically determined (Janssen & Bancroft, 2007). Moreover, theoretical work suggests that CSB could be better conceived as a personality organization, characterized by specific motivations and persistent maladaptiveness of sexual behavior, along with stability and long duration of these patterns (Montaldi, 2003). These pieces of evidence indicate that the CSB network might be already stably structured in adolescence and persist with virtually no change across different age groups. Last, in the adolescent and young adult samples two communities emerged, while in the adult and older samples three communities were detected. It is to note that both two- and three-factor structures for the SCS have been documented (Ballester-Arnal et al., 2013; Liao et al., 2015; Kingston et al., 2018).

Finally, individuals at high and low risk of developing full-blown CSBD were characterized by networks that were similar in terms of structure and global connectivity. Nevertheless, the node reflecting the experience of failing to meet commitments and responsibilities due to one’s sexual behavior (item 4) was statistically more central in at-risk individuals. This finding mirrors a recent cluster analysis study, where neglect (of which the SCS item 4 was the most representative, as expressed by the highest factorial loading) could strongly discriminate between individuals with and without CSBD (Castro-Calvo et al., 2020). Taken together, these pieces of evidence indicate that both magnitude and the specific role of negative consequences due to CSB ought to be considered in order to provide a comprehensive clinical assessment. Future studies should focus on evaluating if specific consequences, such as work-related, personal, health-related, or relationship-related, might have different impacts on mental well-being and psychosocial functioning (Koós et al., 2021).

### Clinical Implications

Two main clinical implications can be derived. First, struggling to control one’s own sexual thoughts and behavior emerged as the most central node of the whole network. While the association between CSB and self-reported impulsivity is often reported (Bőthe et al., 2018; Carvalho et al., 2015), studies relying on behavioral measures of impulsivity provided mixed evidence, with both positive and null association being reported (Carvalho et al., 2021; Miner et al., 2016). Taken together, these findings may indicate that individuals with CSB do not necessarily show an impaired impulse control, but they may be characterized by maladaptive beliefs about the controllability of their sexual behavior (Pachankis et al., 2014). Hence, although equating node centrality to causality is currently debated (Dablander & Hinne, 2019), targeting personal beliefs that sexual behavior is completely out of control could represent a major focus for clinical interventions in individuals with CSB. Accordingly, clinical guidelines, case studies, and empirical work also suggest that building personal self-efficacy and restructuring self-justification cognitions may be an effective way in treating CSB and preventing future relapses (Dilley et al., 2007; Shepherd, 2010; Weiss, 2004).

Second, failing to meet commitments and responsibilities was statistically more central in individuals at high risk than in those at low risk. In other words, in vulnerable individuals CSB-related consequences were not only more negative (i.e., mean), but they also likely played a different role in the network (i.e., centrality). It is possible to speculate that negative consequences may function as important stressors, which lead to negative mood that, in turn, is dealt with by means of compulsive sexual behaviors (Dhuffar et al., 2015). Hence, preventing major negative consequences could represent an effective way to prevent the full-blown disorder (Lew-Starowicz et al., 2020). Moreover, reporting negative consequences due to hypersexuality was the strongest predictor of experiencing the need for professional help in a large, online sample of over 58,000 individuals (van Tuijl et al., 2020). Hence, a comprehensive evaluation of negative consequences due to CSB is definitely advisable, for both treatment and prevention reasons.

### Limitations

Several limitations should be taken into account. First, CSB as described by ICD-11 is a complex phenomenon and it consists of several features, which were not all captured by SCS (e.g., persistence of the excessive sexual behavior despite the absence of satisfaction). While future studies may complement my results with CBSD-specific measures (i.e., Compulsive Sexual Behavior Disorder Scale-19; Bőthe et al., 2020), it is to note the SCS is still one the most frequently used measures for this phenomenon and investigating the structure of SCS is worthwhile. Moreover, a recent meta-analysis reported that it is possible to detect central nodes and robust edges in psychopathology, despite relying on different measures for the same construct (Malgaroli et al., 2021). Second, data were cross-sectional in nature and this prevented from directly estimating the temporal dynamics of CSB within the single individuals and across the different age groups. Future studies should consider relying on experience sampling studies or performing long-term longitudinal studies, which will allow to capture individual-based dynamics and track the developmental trajectory of CSB. Third, in this study only a limited sample of adolescents were included and this might have prevented the detection of small differences with respect to the other groups. Moreover, it was not possible to perform any network estimation on the elderly group, due to insufficient sample. Future studies should aim for a larger recruitment of elderly and adolescents, including also children (Adelson et al., 2012). Despite this limitation, the networks showed good-to-excellent indices of reliability and trustworthiness. In the future, the estimated network should be replicated in a larger sample, using SCS along with other measures for CSB (Hook et al., 2010; Montgomery-Graham, 2017). Fourth, limited information is provided with respect to the sociodemographic characteristics and no information is offered about the cultural background of the participants. By consequence, the degree of representativeness of the sample is difficult to evaluate. Future studies should conduct a population-based, stratified sample recruitment procedure and include additional individual differences, among which sexual orientation and psychiatric assessment. On the other hand, integrating network analysis with cross-validation led to the estimation of a cross-validated predictive network that was markedly similar to that estimated on current data. This indicates that the present results are not biased by overfitting and, in turn, are likely to generalize to future, independent studies. Moreover, performing secondary analysis on open-access data sets is a highly ethical practice, in that, among other benefits, it maximizes the value of public investment in data collection and reduces the burden on respondents (Kievit et al., 2022). Fifth, data collection was performed in 2012. In the future, researchers may want to replicate these results on more recent data and explore whether different sociocultural conditions associated with the passing of time have an impact on the structure of CSB.

### Conclusions

In conclusion, this study shed light on the structure of CSB, as measured with the Sexual Compulsivity Scale, and revealed that impulse dyscontrol could be a major focus of clinical evaluation and perhaps a locus for clinical interventions. Moreover, the network of association among the CSB elements is not likely to be different across different age groups or between males and females. Finally, although no significant structural or connectivity differences were found between individuals at low and high risks, failing to meet one’s commitments and responsibilities was more central in individuals at high risk than in those who are at low risk. This element could be of importance for both preventive and treatment purposes.

## Supplementary Information

Below is the link to the electronic supplementary material.

Supplementary file1 (DOCX 1165 kb)

## Footnotes

1 The data set was uploaded on July 16, 2012, and it was retrieved on November 1, 2021. The participants provided their answer spontaneously and anonymously. At the beginning of the questionnaire, participants were informed that their answer could be used for research purposes. Out of the total sample of individuals who provided their answers, only those who indicated that their results were accurate and suitable for research (79%) were disseminated.
