# Supplementary material for: The Structure of Compulsive Sexual Behavior: A Network Analysis Study
Source: Arch Sex Behav. 2023 Feb 3;52(3):1271–84. doi: 10.1007/s10508-023-02549-y (PMC10102046; doi:10.1007/s10508-023-02549-y)
Supplement: Supplementary file 1 — Supplementary file1 (DOCX 1165 kb) [file 10508_2023_2549_MOESM1_ESM.docx]

**Supplementary Material**

| **Table S1.** Total score, means, standard deviations, and Pearson’s correlations for the total sample (n = 3186). | | | | | | | | | | | | |  | |
| --- | --- | --- | --- | --- | --- | --- | --- | --- | --- | --- | --- | --- | --- | --- |
| Item | *M* | *SD* | 1 | 2 | 3 | 4 | 5 | 6 | 7 | 8 | 9 | 10 | |  |
| 1 | 2.32 | 1.09 |  |  |  |  |  |  |  |  |  |  | |  |
| 2. | 2.24 | 1.07 | .62 |  |  |  |  |  |  |  |  |  | |  |
| 3. | 2.23 | 1.08 | .54 | .68 |  |  |  |  |  |  |  |  | |  |
| 4. | 1.95 | 1.04 | .44 | .54 | .64 |  |  |  |  |  |  |  | |  |
| 5. | 2.24 | 1.12 | .41 | .45 | .53 | .46 |  |  |  |  |  |  | |  |
| 6. | 3.10 | 0.97 | .36 | .32 | .43 | .33 | .46 |  |  |  |  |  | |  |
| 7. | 2.21 | 1.07 | .45 | .54 | .55 | .48 | .58 | .43 |  |  |  |  | |  |
| 8. | 2.30 | 1.09 | .47 | .58 | .58 | .49 | .61 | .44 | .72 |  |  |  | |  |
| 9. | 2.47 | 1.16 | .34 | .45 | .45 | .34 | .41 | .41 | .50 | .58 |  |  | |  |
| 10. | 2.52 | 1.19 | .45 | .35 | .40 | .32 | .39 | .39 | .35 | .37 | .34 |  | |  |
| total score | 23.57 | 7.82 | .71 | .77 | .80 | .70 | .74 | .62 | .78 | .81 | .67 | .62 | |  |
| *Note.* All correlations were statistically significant a *p* < .001 | | | | | | | | | | | | |  | |

| **Table S2.** Total score, mean and standard deviations in males (n = 2175) and females (n = 1011) | | | | | | | |
| --- | --- | --- | --- | --- | --- | --- | --- |
| Item | *M_males_* | *SD_males_* | *M_females_* | *SD_females_* | t-test | *p* | *Cohen’s d* |
| 1 | 2.31 | 1.08 | 2.33 | 1.11 | .31 | ns | .01 |
| 2. | 2.26 | 1.07 | 2.20 | 1.06 | 1.49 | ns | .06 |
| 3. | 2.25 | 1.07 | 2.21 | 1.10 | .81 | ns | .03 |
| 4. | 1.94 | 1.04 | 1.97 | 1.04 | .83 | ns | .03 |
| 5. | 2.17 | 1.10 | 2.40 | 1.14 | 5.40 | < .001 | .21 |
| 6. | 3.08 | 0.95 | 3.15 | 1.00 | 1.86 | ns | .07 |
| 7. | 2.20 | 1.07 | 2.21 | 1.07 | .17 | ns | .01 |
| 8. | 2.29 | 1.08 | 2.32 | 1.09 | .77 | ns | .03 |
| 9. | 2.45 | 1.15 | 2.52 | 1.16 | 1.53 | ns | .06 |
| 10. | 2.55 | 1.18 | 2.46 | 1.22 | 1.94 | ns | .03 |
| total score | 23.49 | 7.68 | 23.76 | 8.12 | .90 | ns | .03 |
| *Note.* Bonferroni-Holm correction was applied | | | | | | | |

| **Table S3***.* Total score, means and standard deviations across age groups, namely adolescents, young adults, adults, and older adults. | | | | | | | | | | | | |
| --- | --- | --- | --- | --- | --- | --- | --- | --- | --- | --- | --- | --- |
| *Item* | *M_ADO_* | *SD_ADO_* | *M_YA_* | *SD_YA_* | *M_ADU_* | *SD_ADU_* | *M_OA_* | *SD_OA_* | *F* | *p* | *η^2^* | *Post-hoc* |
| 1 | 2.02 | 1.08 | 2.21 | 1.06 | 2.41 | 1.09 | 2.39 | 1.10 | 11.35 | <.011 | .011 | ADO, YA < ADU, OA |
| 2. | 2.23 | 1.10 | 2.16 | 1.05 | 2.25 | 1.07 | 2.36 | 1.09 | 5.10 | <.022 | .005 | YA < OA |
| 3. | 2.36 | 1.15 | 2.18 | 1.07 | 2.27 | 1.08 | 2.25 | 1.07 | 2.12 | 1 | .002 |  |
| 4. | 2.06 | 1.09 | 1.97 | 1.03 | 1.95 | 1.04 | 1.87 | 1.04 | 1.67 | 1 | .002 |  |
| 5. | 2.42 | 1.23 | 2.29 | 1.12 | 2.26 | 1.11 | 2.07 | 1.08 | 7.13 | <.011 | .007 | ADO, YA, ADU > OA |
| 6. | 3.18 | 0.97 | 3.10 | 0.95 | 3.12 | 0.96 | 3.04 | 1.01 | 1.16 | 1 | .001 |  |
| 7. | 2.15 | 1.14 | 2.17 | 1.08 | 2.24 | 1.06 | 2.20 | 1.06 | 1.04 | 1 | .001 |  |
| 8. | 2.34 | 1.14 | 2.29 | 1.08 | 2.35 | 1.09 | 2.22 | 1.07 | 1.82 | 1 | .002 |  |
| 9. | 2.47 | 1.20 | 2.50 | 1.16 | 2.46 | 1.16 | 2.42 | 1.14 | .75 | 1 | .001 |  |
| 10. | 2.38 | 1.24 | 2.40 | 1.19 | 2.61 | 1.18 | 2.60 | 1.16 | 7.70 | <.011 | .007 | YA < ADU, OA |
| total score | 23.63 | 8.33 | 23.27 | 7.64 | 23.93 | 7.91 | 23.44 | 7.86 | 1.52 | 1 | .001 |  |
| *Note.* ADO: adolescents; YA: young adults; ADU: adults; OA: older adults. *p*-values were adjusted for Bonferroni-Holm correction. | | | | | | | | | | | | |

| **Table S4.** Means and standard deviations in individuals at low risk (n = 1631) and high risk (n = 1555) | | | | | | | |
| --- | --- | --- | --- | --- | --- | --- | --- |
| Item | *M_lowrisk_* | *SD_lowrisk_* | *M_highrisk_* | *SD_highrisk_* | t-test | *p* | *Cohen’s d* |
| 1 | 1.67 | .78 | 2.99 | .95 | 42.8 | < .001 | 1.52 |
| 2. | 1.55 | .70 | 2.96 | .91 | 49.3 | < .001 | 1.75 |
| 3. | 1.51 | .64 | 3.00 | .91 | 53.8 | < .001 | 1.91 |
| 4. | 1.36 | .60 | 2.56 | 1.04 | 39.9 | < .001 | 1.42 |
| 5. | 1.56 | .75 | 2.95 | .98 | 45.0 | < .001 | 1.60 |
| 6. | 2.62 | .96 | 3.60 | .66 | 33.4 | < .001 | 1.18 |
| 7. | 1.52 | .71 | 2.92 | .89 | 49.1 | < .001 | 1.74 |
| 8. | 1.56 | .71 | 3.08 | .85 | 54.7 | < .001 | 1.94 |
| 9. | 1.83 | .94 | 3.14 | .97 | 38.4 | < .001 | 1.36 |
| 10. | 1.91 | 1.01 | 3.15 | 1.01 | 34.3 | < .001 | 1.22 |
| *Note.* Bonferroni-Holm correction was applied | | | | | | | |

**Figure S1.** Nonparametric bootstrapped confidence intervals of estimated edges. The red line represents the estimated edge, while the dark area indicates the 95% boostrap confidence interval.

**Figure S2.** Nonparametric bootstrapped difference test for the edges. Gray boxes indicate no significant difference, whereas black boxes indicate statistically significant difference (p < 0.05). Diagonal color and saturation represent the magnitude and direction of each estimated edge.

**Figure S3.** Nonparametric bootstrapped difference test for the strength index. Gray boxes indicate no significant difference, whereas black boxes indicate statistically significant difference (p < 0.05). Diagonal values represent the strength score of each node.

**Figure S4.** Network estimated on raw data and cross-validated predicted network

**

**Figure S5.** Strength computed on raw data and based on cross-validation

**Figure S6.** Network visualization by gender.

**

**Figure S7.** Strength scores by gender

**

**Figure S8.** Nonparametric bootstrapped confidence intervals of estimated edges, by gender. The red line represents the estimated edge, while the dark area indicates the 95% boostrap confidence interval.

*
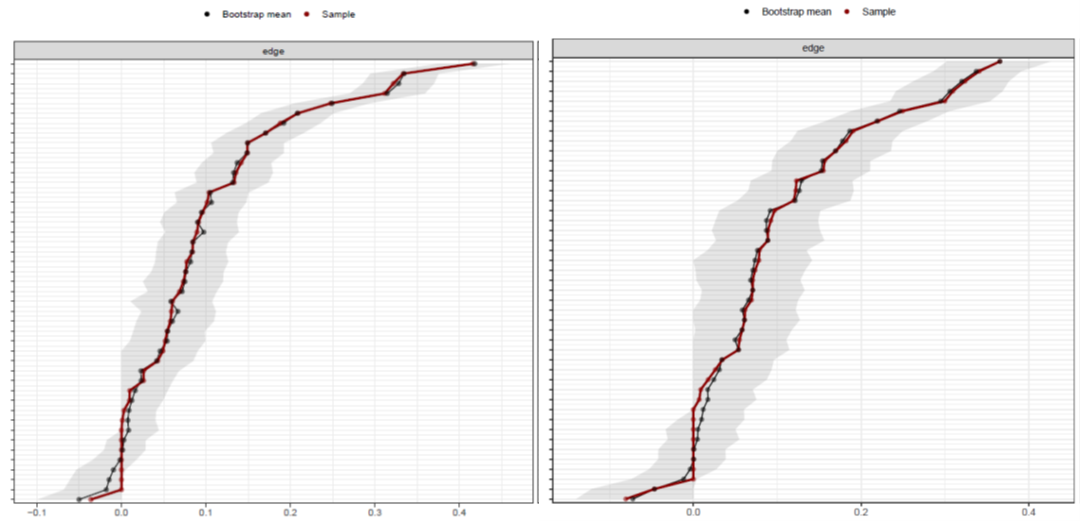
*

**Figure S9.** Nonparametric bootstrapped confidence intervals of estimated edges across the five age groups. The red line represents the estimated edge, while the dark area indicates the 95% boostrap confidence interval.

**Figure S10.** Moderated network of hypersexuality, where age serves as moderator

.

**

**Figure S11.** Network visualization by risk status

.

**Figure S12.** Nonparametric bootstrapped confidence intervals of estimated edges, by risk status. The red line represents the estimated edge, while the dark area indicates the 95% boostrap confidence interval.

*
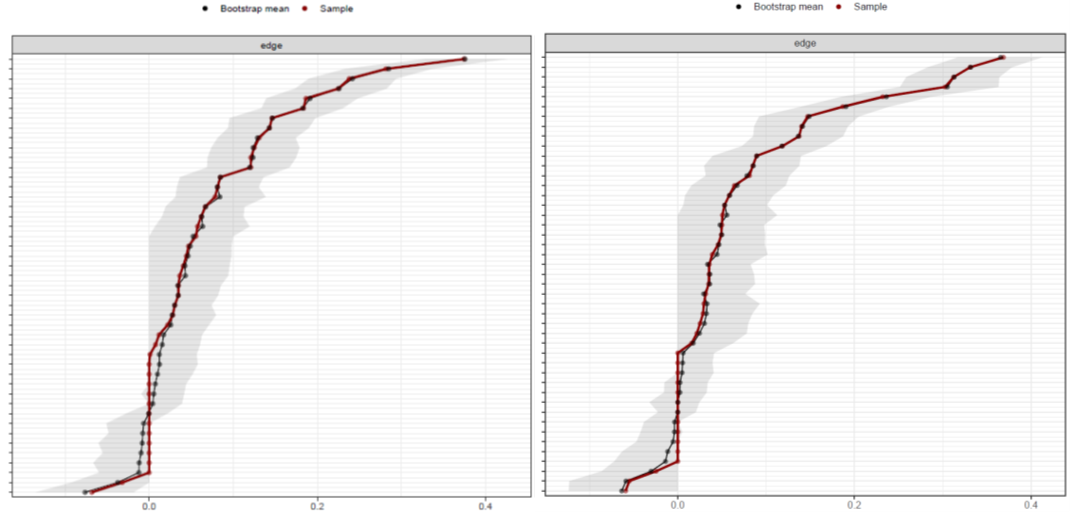
*

**Figure S13.** Strength scores by risk status

**
